# Lineage Tracing of Distal Lung Epithelial Progenitors in Injury‐Induced Regeneration

**DOI:** 10.1111/crj.70143

**Published:** 2025-11-29

**Authors:** Jian Xu, Yuhan Wang, Cuiping Zhang, Xiaoyan Chen, Tianchang Wei, Weiqi Mao, Yuanlin Song

**Affiliations:** ^1^ Department of Pulmonary and Critical Care Medicine Zhongshan Hospital, Fudan University Shanghai China; ^2^ Shanghai Institute of Infectious Disease and Biosecurity, Fudan University Shanghai China; ^3^ Shanghai Respiratory Research Institute Shanghai China; ^4^ National Clinical Research Center for Aging and Medicine Huashan Hospital, Fudan University Shanghai China; ^5^ Key Laboratory of Chemical Injury, Emergency and Critical Medicine of Shanghai Municipal Health Commission, Center of Emergency and Critical Medicine Jinshan Hospital of Fudan University Shanghai China

**Keywords:** epithelial progenitors, lineage tracing, lung injury, regeneration, stem cells

## Abstract

**Background:**

Lineage tracing is an emerging technology with the outstanding advantage of labeling stem cells and their descendants with temporal and spatial specificity in vivo. We aimed to systematically review the research advances of distal lung epithelial progenitors via lineage tracing strategies.

**Results:**

The distal lung, including bronchioles and alveoli, carries the respiratory function and is the central region involved in acute respiratory distress syndrome and other diseases. Many endogenous epithelial stem cell/progenitor lineages such as Club cells, alveolar type II cells, bronchioalveolar stem cells, and basal‐like progenitors that contribute to distal lung regeneration have been identified and are engaged in repairing lung injury for various reasons. Advances in lineage tracing technology have provided tremendous support in characterizing progenitor lineages, identifying new progenitor cell lineages, and discovering regulators of their behaviors.

**Conclusions:**

The important role of distal lung epithelial progenitors and lineage tracing methods has been highlighted in recent years. Relevant studies provide a perspective for further deepening lineage tracing in lung progenitor research and laying the groundwork for endogenous stem cell therapies in the future.

## Graphical abstract

Lineage tracing reveals the diverse stem‐like progenitors driving distal lung regeneration. We highlight how Clara cells, AT2 cells, bronchioalveolar stem cells, basal‐like progenitors, and other progenitors contribute to injury repair through context‐dependent plasticity. Advances in tracing technology not only refine cell fate mapping but also provide insights for future endogenous stem cell–based therapies.

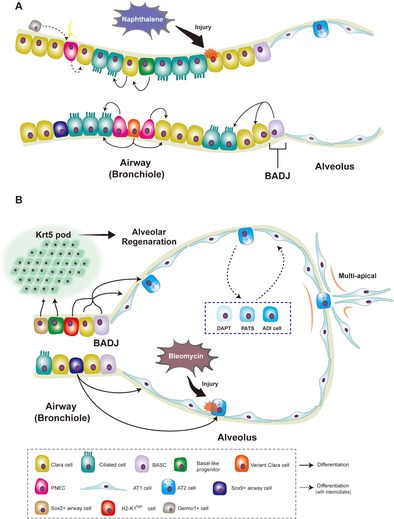

**Abbreviations**
ADI cell: alveolar differentiation intermediate cell
ARDS: acute respiratory distress syndrome
AT1 cell: alveolar type I cell
AT2 cell: alveolar type II cell
BADJ: bronchioalveolar duct junction
BASC: bronchioalveolar stem cell
CCSP: Club cell secretory protein
DASC: distal airway stem cell
DATP: damage‐associated transition progenitor
FGF: fibroblast growth factor
Krt: keratin
LNEP: lineage‐negative epithelial progenitor
NEB: neuroepithelial body
PATS: pre‐alveolar type‐1 transitional cell state
PNEC: pulmonary neuroendocrine cell
Scgb1a1: Secretoglobin family 1 A member 1
SFTPC/SPC: surfactant protein C
Trp63: transformation‐related protein 63

## 1 Introduction

Pulmonary parenchyma consists of different levels of airways and alveoli. Although pulmonary cells are exposed to external damaging agents, they hold a relatively low turnover rate, which is not conducive to the maintenance of normal pulmonary epithelial structures [1, 2]. Thus, the mechanisms of lung injury and regeneration have received considerable attention, among which the investigation of endogenous stem cells or progenitors is pivotal. The distal lung, which includes terminal bronchioles and alveoli, undertakes the function of gas exchange and is threatened by diseases, including interstitial lung disease, cancer, infection, and acute respiratory distress syndrome (ARDS). Now, there is still a need for universally effective therapies for ARDS other than lung‐protective ventilation strategies. Promoting the restoration of distal lung epithelial integrity is a potential avenue for the treatment of ARDS. Studies on epithelial progenitors are expected to provide more insight into ARDS occurrence, development, and treatment.

Lineage tracing is crucial for the investigation of stem cells or progenitors. There exist many widely used tracing systems like inducible Cre‐loxP, Dre‐rox, Flp‐FRT, and Tet system. With the help of specifically expressed recombinase and reporter strains like *Z/AP*, *Z/EG*, *R26‐LacZ*, *mT/mG*, *Brainbow*, and *Confetti*, it is possible to label cell lines in vivo. Briefly, lineage tracing technology utilizes cell‐specific recombinases like Cre to activate a reporter gene by means of inducible gene editing, making the reporter gene a unique marker for stem cells and their progeny. Remarkable progress has been witnessed in lineage tracing in various organs, e.g., the gut, heart, muscle, brain, and vessels. In addition, lineage tracing is also a novel method to study the mechanism of tumorigenesis. The role of lineage tracing in distal lung epithelial studies includes the confirmation of progenitor cell descendants, identification of new progenitor cell subpopulations, and analysis of the transitional state of stem cell differentiation. Recently, more sophisticated tracing strategies have been established. Split‐Cre system and dual lineage tracing approaches have been used to meet various research requirements [3, 4]. A combination of lineage tracing and synthetic Notch signal can even monitor cell–cell contacts and cell fate plasticity in vivo [5].

Progenitor cells with a role in repairing distal lung epithelium include Club cells, alveolar type II (AT2) cells, bronchioalveolar stem cells (BASCs), basal‐like progenitors, etc. BASCs are recently highlighted epithelial progenitors located in the distal lung in mice. Then, some rare basal‐like cells, different from classical basal cells, are also thought to be involved in distal lung regeneration to some extent. In this review, we focus on these four epithelial progenitor lines and discuss the contribution of lineage tracing toward the revelation of these cell lines.

## 2 Hierarchy of Distal Lung Epithelial Cells

The distal lung epithelium is organized as a dynamic cellular hierarchy spanning the airways to the alveoli. In the bronchi and bronchioles, basal cells serve as multipotent progenitors that sustain the pseudostratified airway epithelium, giving rise to secretory Club cells and ciliated cells during homeostasis and repair [6]. Further along the distal airway, the Club cells or basal‐like progenitors maintain the bronchiolar epithelium and, under injury conditions, can exhibit facultative plasticity by adopting “stem‐like” programs to replenish damaged alveoli [7]. Deep in the gas exchange region, AT2 cells function as long‐lived progenitors of the alveolus: they self‐renew and differentiate into the flattened type I (AT1) cells that form the alveolar surface for gas exchange [8]. This classical paradigm of spatially specific progenitor domains (basal, Club, and AT2 cells) maintaining the epithelial hierarchy from airways to alveoli is now further enriched by rare, spatially restricted progenitor populations and transitional states. Notably, at the bronchioalveolar duct junction (BADJ), a subset of cells coexpressing both airway (*Scgb1a1* ^+^) and alveolar (*Sftpc* ^+^) markers has been identified. These cells can self‐renew and bidirectionally differentiate into both bronchiolar and alveolar lineages, constituting a stem cell reservoir at the interface of airways and alveoli [9]. In addition, recent studies have illuminated transient regenerative intermediates that emerge during lung repair. For example, upon alveolar injury, a distinct AT2‐derived transitional cell state (often marked by *Krt8* and other stress markers) appears en route to AT1 cell maturation. These orchestrate regeneration but can aberrantly persist in chronic disease [10]. Likewise, after severe injury that ablates many AT2 cells, airway secretory cells can give rise to a distinct, p63‐expressing progenitor state [7]. These progenitors migrate into the alveoli and ultimately regenerate new AT2 and AT1 cells. Such findings underscore that lung regeneration is not driven by a single immutable alveolar stem cell line, but rather by a repertoire of differentiated cells that become plastic in response to context. Indeed, plasticity is a hallmark of the lung epithelium: cell identity and fate are heavily context‐dependent, with multiple cell types able to transiently assume stem/progenitor functions when prompted by niche signals or injury stress [11]. Consequently, the term “stem‐like cell” is favored over “strict stem cell” in describing distal lung epithelial progenitors, reflecting that many of these cells do not continuously fuel turnover like classic stem cells, but can revert to a progenitor state and regenerate tissue when needed. This flexible, distributed stem cell model sets the stage for the following sections, which will examine four principal cell types, detailing how each contributes uniquely to lung epithelial maintenance and injury repair while interacting within the broader hierarchical network of distal lung regeneration [12].

## 3 Main Epithelial Progenitors in the Distal Lung

### 3.1 Club Cells

Club cell is a type of nonciliated secretory cell in the distal airway. Club cells are potential epithelial cells with stemness contributing to bronchiolar and alveolar cells. Secretoglobin family 1 A member 1 (*Scgb1a1*), also known as CC10, CC16, or CCSP, is a crucial marker of Club cells. *Scgb3a2* and uroplakin 3a (*Upk3a*) have been found in some subsets of Club cells [13]. *CCSP‐rtTA/tetO‐Luc*, *CCSP‐rtTA/tetO‐Cre/ZAP*, and *CCSP‐rtTA/tetO‐Cre/ZEG* strains were established to label Club cells with the help of the Tet system [14, 15]. *CCSP‐Cre* was generated by inserting Cre into exon 1 of *Scgb1a1* [16]. Then, Rawlins et al. [17, 18] created an inducible tracing strain named *Scgb1a1‐CreER*, dominating the research of Club cell tracing afterward. Actually, *Scgb1a1* is also expressed in some mouse AT2 cells [17], which means *Scgb1a1*‐driven tracing techniques may sacrifice a small degree of specificity [16, 19]. Variant Club cells located in the neuroepithelial body (NEB) microenvironments are more injury‐resistant [13, 20]. *Upk3a‐CreER* ^*T2*^ transgenic mouse strain can trace variant Club cells in NEBs [13].

Lineage tracing has verified self‐renewal and differentiation into airway ciliated cells of Club cells. Lineage tracing has also shed light on how Club cells contribute to alveolar cells. AT2 cells could be *Scgb1a1*‐specific tracing positive in bleomycin‐induced lung injury models, indicating *Scgb1a1* ^+^ cells' contributions to alveolar repair [8, 21, 22]. However, divergence exists in the regeneration process of Club cells during oxygen exposure–related injury. Initially, no evidence that *Scgb1a1* ^+^ alveolar cells existed was found in an adult oxygen exposure model [17]. However, exposure to low or high oxygen at birth promoted the differentiation of *Scgb1a1* ^+^ cells to AT2 cells [23]. Another study showed that airway AT2‐like cells in infected mice exposed to neonatal hypoxia or hyperoxia were *Scgb1a1*‐specific labeled [24]. These results may indicate that the stemness of Club cells differs according to the injury conditions. In addition, H2‐K1^high^ cells are a distinct transformation‐related protein 63 (*Trp63* or *p63*) negative Club cell‐like progenitor population accounting for 6% of *Scgb1a1‐CreER* labeled airway cells [25]. Their capability of expansion and differentiation into both airway and alveolar cells was demonstrated both in vitro and in vivo [25]. H2‐K1^high^ cells are also defined as MHC‐II Club cells [26]. More detailed and in‐depth efforts are needed to resolve the puzzle of the role of Club cells in alveolar regeneration.

Effective epithelial repair hinges on a functional balance between Club cells, basal cells, and rare progenitors, with these populations engaging in context‐dependent collaboration to restore tissue integrity. Using *Scgb1a1‐CreER*; *LSL‐YFP*; *CK5‐rtTA*; *tet(O)DTA* mice, it was found that ablation of basal cells made Club cells dedifferentiate into basal stem cells and differentiate into ciliated cells [27, 28]. Earlier studies using the *Scgb1a1‐CreER* lineage tracing system suggested that a majority of *p63* ^+^ cell patches emerging after influenza infection or bleomycin injury were derived from Club cells, indicating a remarkable plastic potential [29]. In bleomycin‐induced alveolar injury, Club cells could dedifferentiate into basal cells and then generate alveolar epithelial cells via fibroblast growth factor 10 (FGF10) signaling [30]. However, more recent lineage tracing studies have clarified that the origin of these *p63* ^+^ progenitors is highly context‐dependent. It is now understood that in influenza (H1N1) infection, many injury‐induced *p63* ^+^ cells actually originate from a preexisting, rare population of *p63* ^+^ *Krt5* ^−^ progenitors, a subset of which can be labeled in the *Scgb1a1‐CreER* system, thus explaining the initial observations [31]. In this model, the contribution of *Scgb1a1*‐lineage cells to the prominent *Krt5* ^+^ pods is now considered limited. In contrast, after bleomycin‐induced alveolar injury, *p63* ^+^ progenitors are indeed generated from airway secretory cells (CC10^+^ cells, Club cells), corroborating the earlier findings [7]. Furthermore, in response to major lung injury, regardless of the model (bleomycin‐induced or influenza infection), repair is predominantly mediated by a preexisting population of *ΔNp63* ^+^ *Krt5* ^−^ cells, later defined as lineage‐negative epithelial progenitors (LNEPs) [32]. In addition, comparative tracing confirms that basal cells, not Club cells, are the major contributors to rare cell types. By comparing the two tracing strains of *Krt5‐CreER* and *Scgb1a1‐CreER,* it was uncovered that basal cells, but not Club cells, have more contributions to some rare cell types like tuft cells, ionocytes, or neuroendocrine cells [33]. Taken together, Club cells, basal cells, and other progenitors are closely interconnected in the repair process.

To sum up, lineage tracing technology has been used in the study of Club cells in multiple dimensions. The specific roles and mechanisms by which Club cells are involved in alveolar repair and their interactions with other progenitor lines can be further explored.

### 3.2 AT2 Cells

AT2 cells are progenitors located in alveoli that maintain alveolar homeostasis and replenish alveolar type I (AT1) cells [34, 35]. Surfactant protein C (SFTPC/SPC) is a component of lung surfactant, which is specific to the lung and mainly expressed in AT2 cells. *Sftpc* has been used as a particular promoter to trace AT2 cells [19]. *SFTPC‐rtTA*, *SFTPC‐rtTA/tetO‐Cre/ZEG*, *SFTPC‐rtTA/tetO‐Cre/ZAP*, and *SFTPC‐rtTA/tetO‐Cre/Rosa26‐YFP* were generated to trace the respiratory epithelium in the developing or mature lung [14, 15, 36]. *Sftpc‐CreER* ^*T2*^ is a popular solution for the inducible tracing of AT2 cells, which is more specific than lineage tracing strategies based on the Tet system [22, 36]. The renewal and differentiation properties of AT2 cells have been confirmed in multiple studies. *Sftpc‐CreER*; *RosaDTA*; *Rosa‐Tm* strain was generated to construct an AT2 cell–specific depletion model [8]. After the targeted depletion of AT2 cells, *Sftpc* ^+^ cells proliferated clonally within 1 week, and AT1 cells only accounted for a small proportion of labeled cells [8]. Using *Sftpc‐CreER* and *LysM‐Cre* tracing strains, it was shown that mature AT2 cells could renew AT1 cells, but could not renew Club, ciliated, and neuroendocrine cells in clonal foci [37]. AT2 cells activated and tripled the number of renewed AT1 cells following hyperoxic injury in 2‐month‐old mice [37]. However, another study found that hypoxia or hyperoxia at birth promoted the self‐renewal of AT2 cells but inhibited their differentiation into AT1 cells [23]. Taken together, lineage tracing techniques are well established for AT2 cell studies, and the reparative effect of AT2 cells on alveoli, but not airways, has been confirmed.

The role of AT2 cells in renewing AT1 cells has been extensively studied. With the invention of AT2‐specific lineage tracing animals, the function and mechanism of AT2 as a progenitor cell of the alveolar epithelium have been further elucidated. As one of the most prominent aspects, the role of subsets of AT2 cells has received considerable attention. Desai et al. [37] revealed that there existed “renewal foci” in perivascular and peripheral regions, contributing to the renewal of alveolar epithelium. They generated the *LysM‐Cre* strain and uncovered that each renewal focus derived from a single, self‐renewing, long‐lived AT2 cell [37]. In *Pseudomonas aeruginosa* –induced lung injury by using *Sftpc*‐specific tracing strategies, the stem cell agent 1 (*Sca1*) positive subset of AT2 cells showed increased proliferation properties in alveolar repair [36, 38]. These *Sca1* ^+^ *Sftpc* ^+^ cells differed from *Sca1* ^−^ *Sftpc* ^+^ cells in proliferation markers [36]. Axis inhibition protein 2 (*Axin2*), a Wnt pathway gene, represented a rare and stable subpopulation of AT2 cells with alveolar progenitor properties in an *Axin2‐CreER* ^*T2*^ tracing manner [39]. After diphtheria toxin–induced lung injury, 73% of AT2 cells started to express *Axin2* [39]. Katsura et al. [40] proposed an inflammatory niche contributed by interleukin‐1 (IL‐1) and tumor necrosis factor α (TNFα), which could enhance adult lung regeneration. After influenza virus infection, surviving AT2 proliferated and contributed to alveolar repair, where IL‐1/nuclear factor kappa‐B (NF‐κB) signaling played an essential role [40]. Corresponding to this study, *Il1r1‐CreER* ^*T2*^; *R26R‐tdTomato* tracing strain was generated, and 15% of AT2 cells were labeled without injury [10]. Bleomycin injury caused the increase of *Il1r1* ^+^ cells and the emergence of labeled AT1 cells at the later phase of injury [10]. Thus, *Il‐1r1* ^+^ AT2 cells could be a distinct subpopulation of AT2 cells responding to injury. Intriguingly, a novel subset of AT2 cells was found just by the level of labeling reporter tdTomato using *Sftpc‐CreER/+*; *tdTomato* ^*flox/flox*^ tracing strain [41]. AT2 subpopulations with low levels of tdTomato (TomLow) expressed lower levels of differentiation markers like *Sftpc* and *Fgfr2b*, whereas AT2 subpopulations with high levels of tdTomato (TomHigh) highly expressed these markers [41]. Pneumonectomy experiments revealed that immature TomLow, but not TomHigh, were progenitors for lung repair [41]. A further study identified TomLow cells as injury‐activated alveolar progenitors (IAAPs), which contribute to alveolar repair during massive depletion of AT2 cells [42].

Various transient cell types have been uncovered to shed light on the exploration of the differentiation process of AT2 cells. Combining lineage tracing with single‐cell RNA sequencing (scRNA‐seq), transitional cell states derived from AT2 cells in bleomycin‐induced lung injury, like primed AT2, cycling AT2, and damage‐associated transition progenitors (DATPs), were identified [10]. DATPs distinctively express markers like *Cldn4*, *Krt8*, and *Ndrg1*, but not typical markers of AT1 or AT2 cells [10]. *Krt8* and *Ndrg1* were used as the specific promoters of lineage tracing for DATPs [10]. The whole study demonstrated an “AT2 cells–DATPs–AT1 cells” differentiation trajectory, in which inflammation signals of IL‐1b and hypoxia‐inducible factor 1α (HIF1a) played an important role [10]. Another study identified the pre‐alveolar type‐1 transitional cell state (PATS), which highly expressed *Cldn4*, *Sox4*, *Lgals3*, and *Fn1*, as a transitional state of AT2 cells [43]. Lineage tracing analysis using *Sftpc‐CreER* and *Krt19‐CreER* strains revealed that PATS originated from AT2 cells and then generated AT1 cells [43]. *Krt8* ^+^ alveolar differentiation intermediate (ADI) cell state was identified in the bleomycin‐induced lung injury model [26]. Lineage tracing analysis based on *Sftpc‐CreER* and *Sox2‐CreER* showed that *Krt8* ^+^ ADI cell state had alveolus/airway dual sources [26]. These transitional cells found by different researchers have similar transcriptional profiles and are all observed in human pulmonary fibrosis [10, 26, 43]. It is reasonable to assume that these transitional cell forms, to some extent, overlap with each other. The discovery of transient cells lays the foundation for directing the organized differentiation of AT2 cells artificially; therefore, treating the alveolar injury.

A cutting‐edge study advanced the knowledge of alveolar regeneration by focusing on the morphological evolution of AT2 cells in lung injury [44]. A single AT2 cell interfaces with multiple alveolar sacs via its multiapical domains. AT2 cells dynamically reestablished their multipolar pattern after specific ablation and tracing using the *Sftpc‐CreER*; *R26R‐DTA*; *R26R‐tdTomato* strain [44]. In the bleomycin‐induced lung injury model, researchers observed that labeled AT1 cells also covered multiple alveolar sacs [44]. Multiapical structures of AT2 cells were destroyed in tumorigenesis in the *Sftpc‐CreER*; *R26R‐LSL‐KrasG12D* strain [44].

Briefly, AT2 cells exhibit characteristics such as having proliferatively active subpopulations and transitional states during differentiation and possessing morphologically suitable properties for injury repair. These features bring essential hints for researching other epithelial progenitor lines.

### 3.3 Bronchioalveolar Stem Cells

BASCs are progenitor populations located in the BADJ that contribute to both bronchial and alveolar repair [45]. Initially, BASCs were considered pollutant‐resistant, BADJ‐associated, and CCSP‐expressing airway progenitor cells [46]. As multipotent progenitors, BASCs were shown to play essential roles in both bronchiolar and alveolar cell injury repair in vivo, and their self‐renewal and multipotency were demonstrated in vitro [9].

**FIGURE 1:**
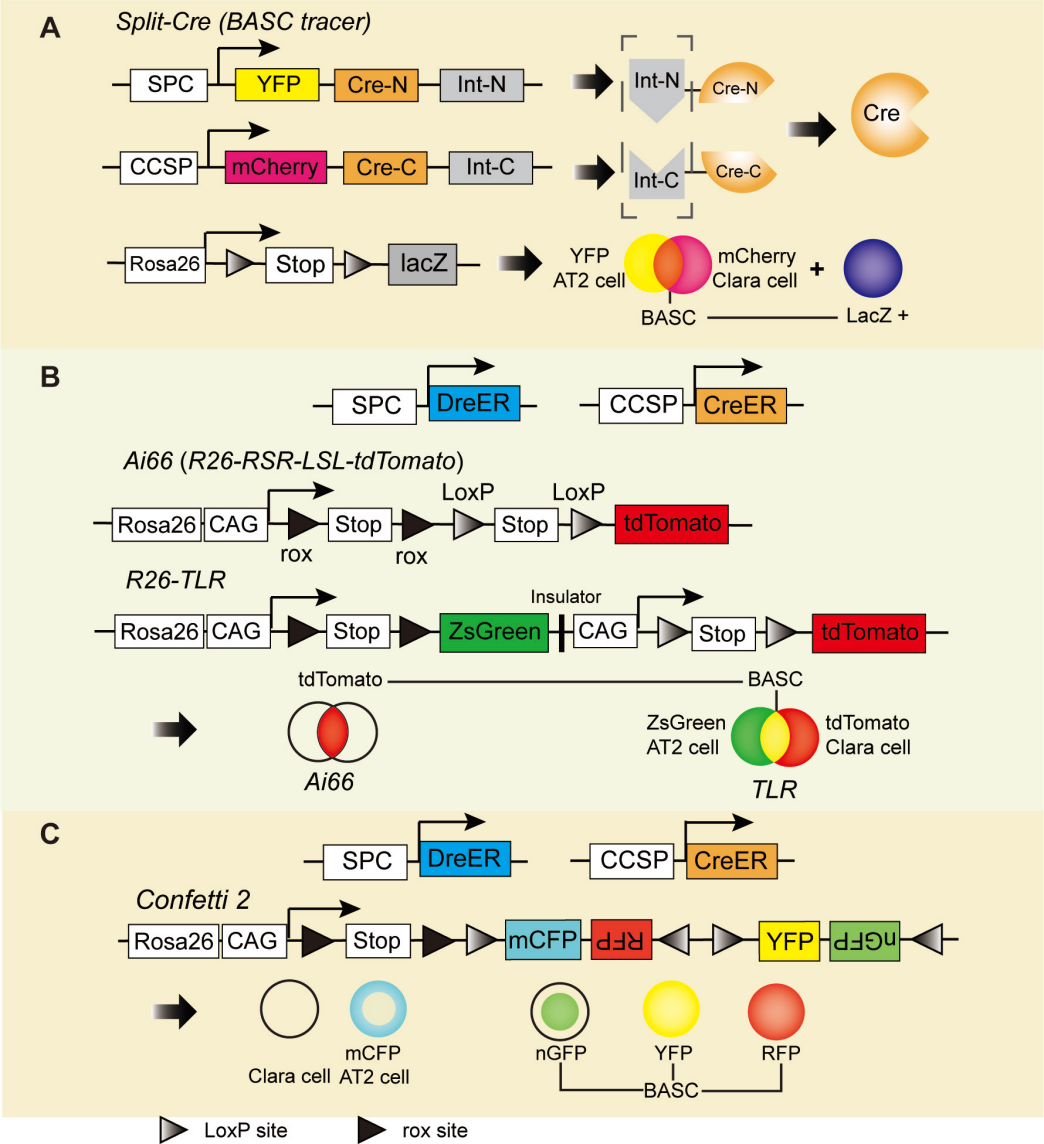
Novel lineage tracing strategies for BASC such as the split‐Cre (*BASC tracer*) (A), dual lineage tracing strategies like *Ai66* and *Rosa26‐TLR* (B), and the *Confetti2* reporter (C). Abbreviations: BASC, bronchioalveolar stem cell; SPC, surfactant protein C; CCSP, Club cell secretory protein; int., intein; N, N‐terminal; C, C‐terminal; GFP, green fluorescent protein; RFP, red fluorescent protein; YFP, yellow fluorescent protein; CFP, cyan fluorescent protein.

The biggest challenge to trace BASCs in vivo is the lack of a single BASC‐specific promoter. CCSP^+^ cells labeled in previous studies actually contained Club cells and a small fraction of BASCs [17, 21], which made it challenging to specifically analyze the properties of BASCs in vivo. BASCs were identified as CCSP^+^ SFTPC^+^ in the lung of normal adult mice by dual immunofluorescence [9]. Using a dual‐specific promoter binding approach, recent BASC tracing studies have provided many innovative and ingenious ways to expand the idea of in vivo studies. Salwig et al. [47] created a split‐Cre‐based BASC tracer and a split‐tTA‐based BASC viewer. As shown in Figure 1A, they split‐Cre or tPA into two nonfunctional halves whose expression was driven by either CCSP or SFTPC, and utilized the split‐intein system, which enabled intein‐mediated protein trans‐splicing to fuse these two halves after their coexistence [47]. Thus, functionally intact Cre or tPA could be induced in BASCs. They also inserted YFP and mCherry reporters into the SFTPC and CCSP loci so that Club and AT2 cells could be labeled simultaneously [47]. They found that BASCs contributed to the bronchioalveolar epithelium slowly in homeostasis and expanded in bronchioles or alveoli after naphthalene or bleomycin‐induced lung injury [47].

Liu et al. [48] crossed *Sftpc‐DreER*, *Scgb1a1‐CreER* and *R26‐RSR‐LSL‐tdTomato* (*Rosa26‐rox‐Stop‐rox‐LoxP‐Stop‐LoxP‐tdTomato*) mice to generate a Cre/Dre dual lineage tracing strain to label BASCs in vivo (Figure 1B). Ideally, the expression of the reporter tdTomato would only occur when both *Sftpc* and *Scgb1a1* are expressed. They found that 77.16 ± 6.12% of dual‐positive cells in BADJ were tdTomato^+^ [48]. They confirmed that BASCs remained stable in number and slowly produced CCSP or SFTPC single‐positive cells in homeostasis [48]. BASCs proliferated and generated Club cells and ciliated cells as descendants in naphthalene‐induced bronchiole injury, whereas AT2 and AT1 cells were replenished by BASCs after bleomycin‐induced alveolar injury, suggesting the multipotency of BASCs [48]. They also used the *R26‐Confetti2* reporter strain (Figure 1C) to trace clones derived from a single BASC, and dual‐positive cells were labeled by RFP, YFP, or GFP [48, 49]. The results were consistent with the above at the single‐cell level [48, 49]. Liu et al. [50] furthered the dual lineage tracing strategy by generating the traffic light reporter (*R26‐TLR*). They induced ZsGreen gene expression in AT2 cells, tdTomato gene expression in Club cells, and both ZsGreen and tdTomato gene expression in BASCs using *Sftpc‐DreER*; *Scgb1a1‐CreER*; *R26‐TLR* mice so that BASCs were labeled in yellow (Figure 1B) [50]. It was shown that most (87.00 ± 1.71%) of ZsGreen+ tdTomato+ cells were located in BADJ regions, consistent with the former studies [50]. The *TLR* reporter has the capability of labeling three progenitor populations in the distal lung concurrently. Their work not only overcame the dilemma of the lack of specific markers for BASCs but also achieved the effect of “1 + 1 > 2”. However, because *Scgb1a1* can also be expressed in AT2 cells [17], the main drawback of these strategies is that some of the putative BASCs can be AT2 cells themselves.

### 3.4 Basal‐Like Progenitors

Basal cells are thought to be the progenitors of large airways. However, in humans, the basal cell–containing pseudostratified epithelium extends distally to the terminal bronchioles, whereas the intermediate bronchus in mice lacks basal cells [51, 52]. This suggests that basal cells may have the capability to repair distal lung regions in human beings as well. Based on the differential distribution of basal cells in human versus murine airways, this review will briefly introduce lineage tracing studies of classical basal cells, with a primary focus on elucidating a related yet distinct cell population crucial for distal lung repair: the intrapulmonary basal‐like progenitors.

The molecular markers of classical basal cells are *Trp63* (*p63*), *Krt5*, *Krt14*, *Ngfr*, *Egfr*, etc. In stratified squamous epithelium, Keratin 5 (*Krt5*) and Keratin 14 (*Krt14*) form the major keratin pair in keratinocytes, and this feature is also present in basal cells. The most popular basal cell–specific lineage tracing promoters are *Krt5* and *Krt14* promoters [19]. The earliest studies used *Krt14‐CreER* ^*T*^ to trace basal cells in lung injury and demonstrated that Club cells and ciliated cells were their descendants [53, 54]. Then, *Krt14*‐expressing cells (K14EC) in the steady state were shown to be able to give rise to Club cells and ciliated cells in a long‐term tracing study [55]. Rock et al. [56] generated the *Krt5‐CreER* ^*T2*^ transgenic tracing strain to monitor basal cells both in homeostasis and injury. Clonal level lineage analysis using *Krt5‐CreER* transgenic strain suggested that *Krt5* ^+^ cells contained two subsets: basal stem cells and basal luminal precursors [57]. $$$$Basal epithelial stem cells (basal ESCs), a newly distinguished AQP3^+^ IL‐33^+^ basal cell subset, participated in bronchiolar–alveolar remodeling in response to influenza infection and expanded by crossing a cell‐growth and survival checkpoint linked to the nuclear‐localized alarmin IL‐33 [58]. *Krt5‐CreER* ^*T2*^; *Il33* ^*fl/fl*^‐mediated basal ESC‐specific ablation showed that the homeostasis in the gut and skin was disrupted, but remodeling and inflammation were downregulated in postviral infection of the lung [58]. These lineage tracing studies demonstrate that classical basal cells predominantly contribute to the repair of airway‐resident cell lineages.

In recent years, some cell lineages with a basal cell phenotype have been identified in studies of distal lung injury models and can renew alveolar cells. This cell population is a rare population of *p63*‐positive cells that reside in the lungs and is commonly designated as basal‐like progenitors. Maria Fernanda de Mello Costa and colleagues [59] have provided a comprehensive review of the alveolar regenerative capacity of basal‐like progenitor cells, highlighting their dual role as a “double‐edged sword” that can lead to maladaptive repair outcomes. Here, we focus on elucidating the pivotal role of lineage tracing techniques in discovering basal‐like progenitor cells and revealing their functional characteristics in lung repair.

Kumar et al. [60] identified *p63* ^+^ *Krt5* ^+^ cells around distal airway tissue as distal airway stem cells (DASCs), which proliferated after H1N1 infection and formed *Krt5* pods in the interstitial lung. In vitro experiments showed that DASCs formed a monolayer structure with occasional CC10 expression in an air–liquid interface model and evolved into alveolus‐like polysphere structures in three‐dimensional Matrigel culture, which could be labeled by alveolus‐specific antibodies [60]. *Krt5* pods derived from DASCs were associated with lung regeneration as evidenced by the evolution of *Krt5* pods into alveolus‐like structures during infection and could be labeled by alveolus‐specific antibodies, although *Krt5* pods were absent in both noninfected and severely infected models [60]. Lineage tracing studies using the *Krt14‐CreER* ^*T*^ strain showed that cells in *Krt5* pods were migrating from the bronchiolar regions, consistent with the positiveness of *Krt6*, an epithelial migration marker, in *Krt5* pods [60]. Further *Krt5*‐specific lineage tracing, *Krt6* ^+^ cell–specific depletion, and transplantation experiments verified the critical role of DASCs in lung regeneration [61]. After H1N1 infection, *Krt5*‐specific traced cells were progressively distributed in the bronchiolar walls and the interstitium [61]. At 60 days postinfection, AT1, AT2, and Club cell markers could be detected in the labeled regions [61]. Initially, it was still believed that this type of progenitor cell expressed the keratin pair KRT5–KRT14, similar to basal cells. Therefore, lineage tracing methods primarily relied on *Krt5/14‐CreER*. Vaughan et al. [32] proposed another *ΔNp63* ^+^ *Krt5* ^−^ cell population called LNEP, which lack mature lineage markers. LNEPs are also a population of progenitors that have the property to proliferate and migrate after severe injury of the distal lung, with the activation and timely shutdown of Notch signaling playing a regulatory role [32, 62]. They also observed that tamoxifen‐induced persistence could potentially interfere with assessing the contribution of Krt5^+^ cells to p63^+^ progenitors [32]. A novel notion they contributed was that under severe injury, with massive depletion of mature cell lines such as AT2 cells, LNEPs are needed to migrate to the injury regions and replenish these cells [32]. Furthermore, *p63‐CreER* ^*T2*^ mice were used to show that *p63* ^+^ LNEPs are the main source of *Krt5* ^+^ cells in both airways and alveoli and infrequently contributed to AT2 cell regeneration [31, 62]. HIF‐1α was demonstrated to be a critical factor in Notch activation and *Krt5* ^+^ cell expansion in hypoxia, although activation of the Wnt signaling or suppression of HIF1α promoted the functional regeneration of AT2 cells on the contrary, indicating the intricate interactions of signaling pathways in LNEP‐mediated lung repair [62]. Thus, there are two contradictory routes for LNEPs to differentiate into *Krt5* ^+^ cells or alveolar epithelial cells, and the counterbalance between HIF1α‐Notch and Wnt signaling may be critical for cell fate.

This series of studies of DASC and LNEP showed that the differentiation route into alveolar cells was mediated by *Krt5* pods to some extent. Several other studies have discussed this process and the origin of *Krt5* ^+^ cells. *Trp63* ^+^ cells appeared in alveolar ducts during the exudative stage in patients with diffuse alveolar damage (DAD) [63]. Ficial et al. [64] found that *p63* ^+^ *Krt5* ^+^ basal cells were usually located within bronchioles in humans, and *Krt14* ^+^ cells increased in alveolar epithelial cells after DAD. However, no evidence of the existence of *ΔNp63* ^+^/*Krt5* ^+^ pods in human lungs was found through biopsy or autopsy [64]. In addition to the basal‐like progenitors mentioned above, Club cells have also been explored as a potential source component. Initially, *Krt5* or *Scgb1a1*‐specific lineage tracing indicated that most of the newly induced *p63* ^+^ cells were derived from Club cells but not preexisting *p63* ^+^ cells [29]. Subsequent lineage tracing experiments based on *Scgb1a1* reported that Club cells contribute minimally to the *Krt5* ^+^ pod [32, 65], although the contribution rates calculated using similar protocols varied across different experimental contexts [31]. Moreover, using multiple lineage tracing strategies, *Sox2* ^+^ lineage‐negative progenitor cells were shown to be the main source of emerging *Krt5* ^+^ cells in influenza‐induced lung injury, whereas *Sox2* ^+^ *Krt5* ^+^ resident basal cells made a significantly minor contribution [65]. Although these studies differ in their understanding of the cellular composition of the *Krt5* ^+^ pod, they collectively reveal the complexity of the lung regeneration system. The very existence of *Krt5* ^+^ cells provides a deeper perspective for our understanding of alveolar regeneration.

It should be noted that the repair process from *p63* ^+^ cells to the *Krt5* ^+^ pod remains limited in its capability to regenerate normal, gas exchange–competent alveolar tissue, and its efficacy may vary across different injury models [59]. For example, the aforementioned study by Vaughan et al. [32], using *Krt5* lineage tracing, found that ~30% of the transitional *Krt5* ^+^ cells eventually acquired AT2 cell characteristics. As a key marker of basal‐like progenitor cells, *ΔNp63* may actually act in a “negative role” by suppressing the recovery of functional alveolar tissue, potentially through silencing the expression of critical markers [66]. The potential of these basal‐like progenitor cells in alveolar repair is fascinating. Nevertheless, current lineage tracing methodologies predominantly revolve around specific markers (e.g., *p63*/*Krt5*). Future research urgently needs to delve into the molecular mechanisms downstream of *p63* to delineate the regulatory patterns that distinguish “beneficial” from “detrimental” repair.

### 3.5 Other Rare/Putative Progenitors

Pulmonary neuroendocrine cells (PNECs) are a relatively small cell population considered both progenitor cells and progenitor cell niches [67]. Achaete‐Scute homologue‐1 (*Ascl1*) is essential for the differentiation of PNECs [68]. *Shh‐Cre*, *Nkx2.1‐Cre*, and *Sox9‐Cre* strains were used to show the homology among PNECs and other respiratory epithelial cells [69, 70]. *Ascl1*‐defined cells (ASDCs) contribute to Club cells in postnatal naphthalene injury, while they give rise to multiple types of airway and alveolar cells during embryonic development [71]. The process of “slithering” in NEB progenitors may be associated with the metastasis of PNECs and cells in small cell lung cancer (SCLC) [69]. A tracing study using *Ascl1‐CreER* further indicated that rare neuroendocrine progenitors possess the capacity for proliferation and migration [72]. Notch2 was enriched in neuroendocrine progenitors, and the activation of Notch signaling was shown to be related to their stemness and transdifferentiation [72, 73]. Calcitonin gene–related peptide (CGRP) is one kind of peptide stored in NEBs. Another tracing strain, *CGRP‐CreER*, could only label PNECs after 15.5 days postcoitum, and their contribution to Club cells and ciliated cells was confirmed in naphthalene‐induced lung injury, but selective ablation of PNECs did not affect Club cell regeneration [70].

Lineage tracing makes it possible to identify some rare progenitor populations. During lung development, *Sox2* and *Sox9*, respectively, mark the proximal and distal epithelial progenitors. *Sox2*‐specific labeled cells made a significant contribution to *Krt5* pods and then replenished alveolar cells [65]. A *Sox9‐CreER* tracing study identified *Sox9*‐expressing cells that played an important role in lung regeneration and radiation repair via the PI3K/AKT pathway [74]. The expression of other stem cell markers like *Sox2*, *Krt14*, *Krt5*, and *p63* had almost no difference between *Sox9* ^−^ and *Sox9* ^+^ cells, indicating the novelty of *Sox9*‐expressing cells [74]. A lineage tracing study using the *Dermo1‐Cre* strain showed that *Dermo1* ^+^ pulmonary mesenchymal cells participated in airway epithelial regeneration [75]. In naphthalene‐induced lung injury, *Dermo1* ^+^ cells contributed to Club and ciliated cells via transitional differentiation into neuroendocrine cells [75]. Further detailed studies are needed to confirm that the above cells are bona fide stem cell or progenitor populations.

## 4 Regulatory Factors of Progenitor Differentiation

A fundamental question in stem cell biology concerns the identity and mechanism of action of the regulators that govern progenitor cell regeneration and differentiation. Intracellular regulators, intercellular interactions, and other external signals provide important perspectives to uncover this issue. Lineage tracing provides noteworthy technical support for the above processes.

Intracellular signals are important factors that regulate cellular activity and mediate intercellular interactions. Wnt, Notch, JAK/STAT, and Hippo/Yap pathways play essential roles in the proliferation and differentiation of stem cells or progenitors. Hippo/yes‐associated protein 1 (*Yap1*) positive cells were mainly *Sftpc*‐specific labeled AT2 cells after lung injury [76], although Hippo pathway‐related promoters have not been used to generate AT2 cell–specific tracing strains. In the *Pofut1* ^*F/F*^; *Tgfb3‐Cre* model, where Notch signaling is inactivated, the *Tgfb3‐Cre* driver labeled approximately 90% of CCSP^+^ cells, concurrent with a phenotype of goblet cell metaplasia and a reduction in Club cells [77, 78]. *Scgb1a1‐CreER*; *LSL‐YFP*; *RBPJk* ^*fl/fl*^ and *Scgb1a1‐CreER*; *LSL‐YFP*; *Notch2* ^*fl/fl*^ mice were generated to show that tonic Notch activation is required to maintain Club cell fate in a quiescent state [27, 28]. Notch2 tension is essential for inhibiting Club cells' differentiation [28]. What's more, Forkhead box protein M1 (*Foxm1*) deficiency caused the ectopic emergence of *Scgb1a1*‐labeled AT2 cells and squamous cells in bronchioles, indicating that *Foxm1* could be a critical transcription factor for normal proliferation and differentiation of Club cells [79]. *Foxm1* is also an essential transcription factor of the highly active *Sca1* ^+^ subset of AT2 cells [29, 38]. Transcription factor grainyhead transcription factor cellular promoter 2‐like 1(*Tfcp2l1*) has been identified as a crucial transcriptional regulator of AT2 cell self‐renewal and differentiation, especially in response to inflammatory signaling [80]. It is evident that signaling pathways and their downstream transcription factors play a regulatory role in the stem cell or progenitor fate, often exhibiting conserved functions across cell types.

Intercellular interactions manifest as stem cell niche effects and paracrine effects. First of all, the maintenance of cell fate requires a specialized microenvironment called the stem cell niche. Interestingly, some stem cells or progenitors play this role themselves. As explained above, Club cells may have a close relationship with basal cells and basal‐like progenitors. Direct contact with basal stem cells is verified to be sufficient to prevent airway secretory cells from both dedifferentiation and differentiation [27, 28]. PNECs are considered progenitor niches. NEBs are essential niches of variant Club cells. The variant Club cells would be ablated in mice lacking PNECs [13]. The essence of specialized stem cell niche formation may be dependent on some contact signals like Notch signaling. Zhang et al. [5] modified the lineage tracing technique to activate the intracellular gene editing system with synthetic Notch proteins, making it possible to construct a cellular communication map. Secondly, paracrine factors also make an important contribution. For example, FGF family epithelial and mesenchymal interactions, with the FGF7 subfamily influencing epithelial cell survival, repair, and differentiation [81]. Deleting FGF receptor 2b (*Fgfr2b*) with *Sftpc* ^*CreERT2*^; *Fgfr2b* ^*flox/flox*^; *tdTomato* ^*flox/flox*^ mice caused the shrinking of AT2 cell populations in homeostasis [42]. Then, in the injury state, FGF10 regulated Club cells' repair of the alveolar epithelium [30]. Therefore, clarifying the role of mesenchymal cell paracrine factors is beneficial for further understanding of the functional processes of epithelial stem cells or progenitors.

The broadest category of regulation encompasses systemic and environmental cues—including external injury, inflammatory responses, and endocrine signaling—which, while ultimately mediated through local cellular pathways, provide diverse perspectives for interrogating stem cell activity. As mentioned previously, the function of Club cells to repair alveoli appears to be affected by the form of lung injury. Take AT2 cells as a detailed example; inflammatory factors like IL‐1β promoted the transformation of AT2 cells to DATPs, which seemed to show a positive impact on regeneration [10]. However, sustained inflammatory signaling led to the accumulation of DATPs and prevented the maturation of AT1 cells [10]. Hence, it is reasonable to explore the regulatory chains and their relationship with stem cell/progenitor behavior in different diseases, different forms of injury, and different phases of time.

## 5 Conclusions and Perspectives

**FIGURE 2:**
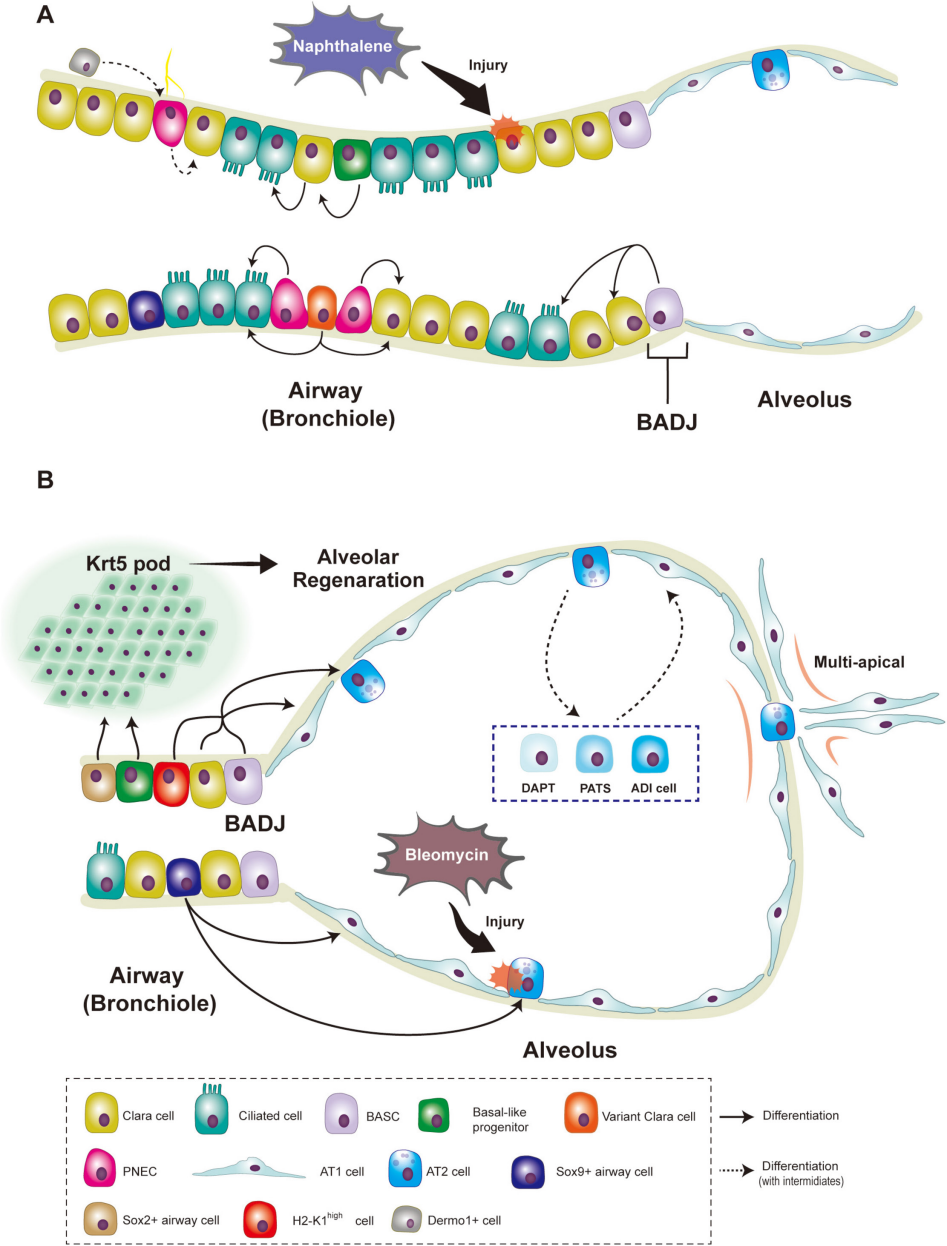
The main differentiation trajectory and regeneration processes via the epithelial progenitors of the bronchiole (A) and alveolus (B). Abbreviations: BASC, bronchioalveolar stem cell; BADJ, bronchioalveolar duct junction; AT1 cell, alveolar type I cell; AT2 cell, alveolar type II cell; PNEC, pulmonary neuroendocrine cell; DASC, distal airway stem cell; LNEP, lineage‐negative epithelial progenitor.

Lineage tracing plays a vital role in the study of postinjury regeneration of the distal lung. The main cell lineages located in the distal lung epithelium are shown in Table 1. As for progenitor lineages, Table 2 lists their predominant tracing strains, and the differentiation trajectory of the bronchiole and alveolus is briefly reviewed in Figure 2.

**TABLE 1:** Main epithelial cell lineages in the distal lung.

Cell lines	Location	Markers	Descendant cells	Tracing strains
Progenitor lines				
Club cell	Airway (mainly bronchioles in humans)	*Scgb1a1*, *Scgb3a2*, *Upk3a*, *Cyp2f2*	Club cell, ciliated cell, basal cell, AT1 cell, AT2 cell	*CCSP‐rtTA* *CCSP‐Cre* *Scgb1a1‐CreER* *Upk3a‐CreER* ^*T2*^
AT2 cell	Alveolus	*Sftpc*	AT2 cell, AT1 cell	*Sftpc‐rtTA* *Sftpc‐CreER* ^*T2*^ *LysM‐Cre* *Axin2‐CreER* ^*T2*^ *Il1r1‐CreER* ^*T2*^
BASC	BADJ	Both *Scgb1a1* and *Sftpc*	BASC, AT1 cell, AT2 cell, Club cell, ciliated cell	*Scgb1a1‐CreER* *BASC tracer* *BASC viewer* *BASC v‐race* *Sftpc‐DreER; Scgb1a1‐CreER; Ai66* *Sftpc‐DreER; Scgb1a1‐CreER; TLR* *Sftpc‐DreER; Scgb1a1‐CreER; Confetti2*
Basal‐like progenitors (DASC/LNEP)	Terminal bronchioles	*p63* or *ΔNp63* (main marker), *Krt5, Krt14* (some lines)	Club cell, Goblet cell, AT1 cell, AT2 cell	*p63‐CreER* ^*T2*^ *Krt5‐CreER* ^*T2*^ *Krt14‐CreER* ^*T*^
PNEC	Airway, NEB	*CGRP*, *Ascl1*, *Calca*	PNEC, Club cell, ciliated cell	*Ascl1‐Cre* *Ascl1‐CreER* ^*T2*^ *CGRP‐CreER* ^*T2*^
Nonprogenitor lines				
Ciliated cell	Airway	*Foxj1*, *Tub‐b4a*	Terminally differentiated	*Foxj1‐Cre,* *Foxj1‐CreER* ^*T2*^
AT1 cell	Alveolus	*Hopx*, *Ager*, *Aqp5*, *Pdpn*	Terminally differentiated	*Aqp5‐Cre* *Hoxp‐CreER* *Hopx‐FlpoER* ^*T*^ *Igfbp2‐CreER* *Ager‐CreER* ^*T2*^

Abbreviations: AT1 cell, alveolar type I cell; AT2 cell, alveolar type II cell; BASC, bronchioalveolar stem cell; DASC, distal airway stem cell; LNEP, lineage‐negative epithelial progenitor; PNEC, pulmonary neuroendocrine cell.

**TABLE 2:** Lineage tracing strains of distal lung epithelial progenitors.

Tracing strain	Description	Ref.
Club cell		
*CCSP‐rtTA*	Used in combination with *tetO‐Luc* or *tetO‐Cre* operator strains.	[14, 15]
*CCSP‐Cre*		[16]
*Scgb1a1‐CreER*	The most widely used Club cell tracing system with a small subset of AT2 cells labeled. Trace subpopulations of Club cells like variant Club cells and H2‐K1 high cells.	[13, 17, 25]
*Upk3a‐CreER* ^*T2*^	Label variant Club cells located in NEBs.	[13]
AT2 cell		
*Sftpc‐rtTA*	Need to be crossed with *tetO‐Luc* or *tetO‐Cre* operator strains.	[14, 15, 36]
*Sftpc‐CreER* ^*T2*^	The most widely used AT2 cell–specific tracing system.	[22, 36]
*LysM‐Cre*	Cre recombinase knock‐in at the LysozymeM (Lyz2) locus. Trace AT2 cells in the embryo but not AT1 cells.	[37]
*Axin2‐CreER* ^*T2*^	*Axin* is a Wnt pathway gene. Label a rare and stable subpopulation of AT2 cells with progenitor capability.	[38]
*Il1r1‐CreER* ^*T2*^	Label a subpopulation of AT2 cells with progenitor capability in response to an inflammatory IL‐1 signal.	[10]
Transitional cell related to AT2 cell		
*Krt8‐CreER* ^*T2*^	Label the damage‐associated transition progenitors (DATPs) that appeared in the differentiation process of AT2 cells.	[10]
*Ndrg1‐CreER* ^*T2*^		[10]
*Krt19‐CreER*	Label the pre‐alveolar type‐1 transitional cells (PATSs) that appeared in the differentiation process of AT2 cells.	[43]
BASC		
*Scgb1a1‐CreER*	Not specific.	[17, 21]
*BASC tracer*	Split‐Cre system driven by both CCSP and SFTPC.	[47]
*BASC viewer*	Split‐tPA system driven by both CCSP and SFTPC. The tetO operator is bidirectional.	[47]
*BASC v‐race*	Based on the split‐tPA system. The expression of Cre is induced by the tPA‐tetO combination.	[47]
*Sftpc‐DreER; Scgb1a1‐CreER; Ai66*	Specific dual lineage tracing strategy for BASCs with the combination of Dre‐rox and Cre‐LoxP systems.	[48]
*Sftpc‐DreER; Scgb1a1‐CreER; TLR*	Label Club cells, AT2 cells and BASCs simultaneously using a dual lineage tracing system.	[50]
*Sftpc‐DreER; Scgb1a1‐CreER; Confetti2*	Label clones derived from a single BASC. BASCs are labeled randomly with one of three luciferases.	[48, 49]
Basal‐like progenitors		
*Krt5‐CreER* ^*T2*^	Widely used basal cell–specific tracing system. Label basal cells both in homeostasis and injury. Label *Krt5* ^+^ DASC but not *Krt5* ^−^ LNEP.	[32, 56, 57]
*Krt14‐CreER* ^*T*^	Label *Krt14*‐expressing cells with stem cell capability. Trace the formation of *Krt5* pods.	[53, 54, 55, 60]
*p63‐CreER* ^*T2*^	Label *p63* ^+^ basal‐like progenitors in the pulmonary region. Label LNEPs more effectively than *Krt5‐CreER* ^*T2*^.	[31, 62]
PNEC		
*Ascl1‐Cre*	Label *Ascl1*‐defined cells (ASDCs) during lung development.	[71]
*Ascl1‐CreER* ^*T2*^	Label PNEC in adult lungs. *Ascl1* or CGRP exists in other nervous tissues.	[71]
*CGRP‐CreER* ^*T2*^		[70]
Other cell lines		
*Sox2‐CreER*	*Sox2* marks proximal airway cells in lung development. Label rare *Sox2* ^+^ progenitors that generate *Krt5* ^+^ cells in *Krt5* pods.	[72]
*Sox9‐CreER*	*Sox9* marks distal airway cells in lung development. Label a putative, novel Sox9^+^ progenitor line.	[74]
*Dermo1‐Cre*	Label *Dermo1* ^+^ pulmonary mesenchymal cells, which participate in bronchiole repair.	[75]

Abbreviations: AT2 cell, alveolar type II cell; BASC, bronchioalveolar stem cell; CCSP, Club cell secretory protein; DASC, distal airway stem cell; IL‐1, interleukin 1; LNEP, lineage‐negative epithelial progenitor; NEB, neuroepithelial body; PNEC, pulmonary neuroendocrine cell; SFTPC, surfactant protein C.

Dual lineage tracing strategies have been utilized in BASC‐specific tracing with the combination of two distinct recombinase systems [48, 50]. For more accurate tracing of stem cells, reporter strains like *Confetti*, *Confetti2*, interleaved reporters, and nested reporters [3] have been designed. Moreover, Cre recombinase is “multifunctional” because the specific expression of Cre can not only mediate lineage tracing but also delete genes of interest [10] or induce other gene expressions [24, 28, 44]. The specific expression of diphtheria toxin A subunit (DTA) can be used to deplete the targeted cell lines. Now, the most pressing need is to improve the resolution and pursue finer conclusions at the single‐cell level. Reporters like *Confetti* and *Confetti2* make it possible to study single‐cell–derived colons. With more sophisticated and well‐designed gene editing systems, single‐cell–lineage tracing is becoming even more available and accessible. Further studies can be aimed at precisely describing lung epithelial progenitor populations, searching for subpopulations with certain progenitor characteristics and gaining insight into their evolution in regeneration.

By reviewing the literature, we conclude that Club cells, AT2 cells, BASCs, and basal‐like progenitors are progenitor populations of great significance in distal lung regeneration. The distal lung epithelium is poised to mount rapid repair responses upon injury. Consequently, lung repair cannot be attributed to a single progenitor population. It is necessary to further elucidate the responses of different progenitor populations to injury of different regions, forms, and degrees. BASCs have attracted new research interest because of their pluripotent properties committed to both bronchiolar and alveolar cells. Whether BASCs exist in the human lung is still under debate. BASCs may correspond to particular subpopulations of human Club cells or AT2 cells. Another issue that needs to be addressed is the intracellular and extracellular regulation of progenitors and the commonality among different progenitor populations. Searching for regulatory commonalities in intracellular, intercellular, and other dimensions could provide a basis for developing stem cell therapies. We conclude that investigating endogenous stem and progenitor cells holds significant promise for developing novel therapies for ARDS and other diffuse lung injury–related diseases.

## Author Contributions

**Jian Xu:** design of the review, literature search, preparation of the manuscript, writing – original draft. **Yuhan Wang:** design of the review, literature search, preparation of the manuscript, writing – original draft. **Cuiping Zhang:** editing of the manuscript, corrections, writing – review and editing. **Xiaoyan Chen:** editing of the manuscript, corrections, writing – review and editing. **Tianchang Wei:** editing of the manuscript, corrections, writing – review and editing. **Yuanlin Song:** supervision, scientific concept and insight, review of the manuscript, writing – review and editing.

## Funding

The authors have nothing to report.

## Ethics Statement

The authors have nothing to report.

## Conflicts of Interest

The authors declare no conflicts of interest.

## Funding

This study was supported by the National Natural Science Foundation of China (82130001, 82272243, 82500110), the Shanghai Three‐Year Action Plan to Strengthen the Construction of the Public Health System (GWVI‐11.1‐18), the National Key Research and Development Program of China (2024YFC3044400), the R&D Program of Guangzhou National Laboratory (GZNL2024A02003), the Shanghai Municipal Science and Technology Major Project (ZD2021CY001), the Science and Technology Commission of Shanghai Municipality (22Y11900800), the Shanghai Municipal Key Clinical Specialty (shslczdzk02201), the China Postdoctoral Science Foundation (2024 M750545), and the Outstanding Resident Clinical Postdoctoral Program of Zhongshan Hospital Affiliated to Fudan University (2025ZYYS‐004).

## Data Availability

No additional data available.
